# Dublin Hareport: The movement ecology and airfield interactions of resident, airside hares, at an international airport

**DOI:** 10.1002/ece3.11490

**Published:** 2024-05-30

**Authors:** Samantha Ball, Anthony Caravaggi, Gerry Keogh, Fidelma Butler

**Affiliations:** ^1^ School of Biological, Earth and Environmental Science, Distillery Fields University College Cork Cork Ireland; ^2^ Dublin Airport Airport Fire and Rescue Service Dublin Ireland; ^3^ Department of Natural Sciences Atlantic Technologic Marine & Freshwater Research Centre Galway Ireland; ^4^ School of Biological and Forensic Sciences University of South Wales Pontypridd UK

**Keywords:** airfield ecology, GPS telemetry, human‐wildlife conflict, movement ecology

## Abstract

Understanding how animals move and use space within an environment is vital for the development and implementation of effective management actions. Within airfield environments, animal movement can present a substantial risk to aircraft, resulting in wildlife‐aircraft collisions (strikes) if animals enter into the manoeuvring areas of the airfield, namely the runways, taxiways and areas that connect the two (hereafter collectively referred to as ‘tarmacked areas’). However, reliable ecological data to inform management decisions can be difficult to obtain in such environments, due to access restrictions. Here, we present the first GPS data describing the movement ecology and spatial use of mammals on an airfield – Irish hare (*Lepus timidus hibernicus)*, at Dublin International Airport – through the deployment of five GPS collars. A total of 4571 tarmacked area interactions were recorded between December 2021 and August 2022, with all five hares engaging with tarmacked areas. Between December and August, the highest number of interactions were recorded for the month of April (*n* = 1073), followed by March (*n* = 703). There was a mean of 4.3 (range: 0–65) interactions with tarmacked areas, per hare, per day throughout the study period. Hares most frequently engaged with tarmacked areas between 05:00 and 07:59, with some seasonal variation. The greatest cumulative distance moved per month was observed in May (505 km) and April (503 km). We identified that the average home range size of collared hares was 2.8 km^2^ (±SD 0.1 km^2^), based on 95% Kernel Utilisation Distribution. Furthermore, we demonstrate that the hares incorporate tarmacked area habitat types into their home ranges with up to 13% of one individual's movements incorporating these areas. Our study demonstrates the suitability of GPS tracking devices for studying the movement ecology of high‐risk mammal species at airfields in order to inform airside management practices.

## Graphical abstract

Here, we present the first GPS data describing the movement ecology and spatial use of mammals on an airfield, using the Irish hare (Lepus timidus hibernicus), at Dublin International Airport, through the deployment of five GPS collars. A total of 4571 active area interactions were recorded between December 2021 and August 2022, with all five hares engaging with manoeuvring areas. This research has important implications for wildlife hazard management.

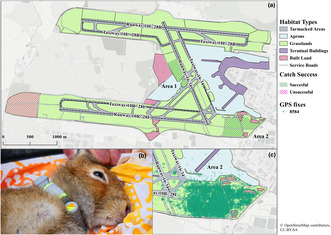

## 1 INTRODUCTION

The expansion of animal movement ecology research, through technological advances, allows for fine‐scale spatial and temporal resolution data to be collected via Global Positioning System (GPS) enabled devices. The use of such devices allows for a more comprehensive understanding of a species' ecology and the practical application of conservation efforts (Chan et al., 2022). Such devices also help document the impacts of anthropogenic disturbance on wildlife, affecting fundamental components of species' life histories such as distribution (Voigt et al., 2020), habitat selection (Suraci et al., 2019) and activity patterns (Parra‐Torres et al., 2020). As an increasing number of species and populations are required to live within anthropogenically modified landscapes, the potential for human‐wildlife conflict arises. These conflicts can range from the spread of zoonotic diseases (Dias et al., 2020) to the destruction of crops (Corriveau et al., 2022) and the occurrence of wildlife‐vehicle collisions (Serieys et al., 2021). Hence, acquiring high quality wildlife movement data within anthropogenically modified landscapes, with the goal of mitigating impacts and conflicts, is a central objective for wildlife managers (Dias et al., 2020).

Airfield environments are one such anthropogenically modified landscape where the opportunity for human‐wildlife conflict arises, due to the occurrence of wildlife‐aircraft collisions, or ‘strikes’. Strike events can be hazardous to passenger safety, with ~350 fatalities reported for civil aviation as a result of strike events since 1912 (Avisure, 2022). Strikes can also result in economic loss (Dolbeer et al., 2023) and be detrimental to the wildlife species involved. While the majority of strikes involve avian species (e.g., 95% in USA; Dolbeer et al., 2023), mammal strikes do occur, and are indeed becoming increasingly common (Ball, Caravaggi, & Butler, 2021). As terrestrial mammals are only a strike hazard to aircraft when entering into parts of the airfield where aircraft are manoeuvring (i.e. runways/taxiways/ connecting areas), understanding the movement patterns and spatial use of species that live on, or near, airfields may aid in strike mitigation efforts.

The complex nature of airfield environments, coupled with the elusive nature of many mammal species, can make the collection of high‐quality ecological data complicated. Hence, researchers often rely on remote sensing methods, such as camera traps (Ball et al., 2022; Carswell et al., 2021). While GPS transmitters have been used to study the movements of large waders (Anatidae) in proximity to airfields (Askren et al., 2019; Lehrke et al., 2017), here we present the first known study to track the movements of a terrestrial mammal, the Irish hare (*Lepus timidus hibernicus*), within an airfield environment. By tracking the movements of a resident population of Irish hare at Dublin International Airport, we aim to (i) understand the frequency in which hares engage with tarmacked areas at the airfield, (ii) identify temporal patterns to tarmacked area interactions, and (iii) identify the size and habitat composition of Irish hare home ranges at Dublin Airport.

## 2 METHODS

### 2.1 Study area

**FIGURE 1:**
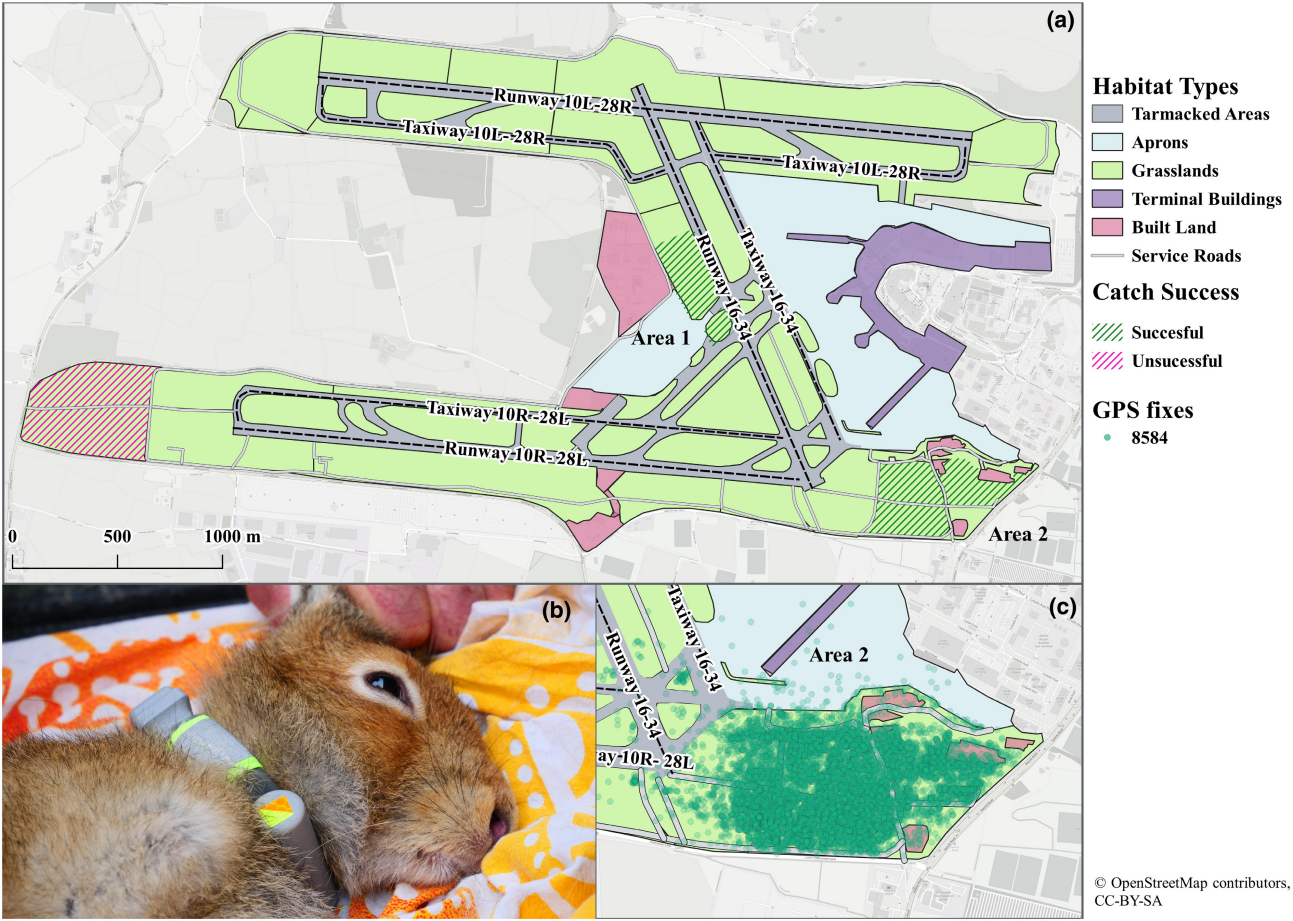
(a) Airside habitat map at Dublin Airport and location of all grasslands where catch attempts were permitted by Air Traffic Control (ATC) and daa. Successful catch areas where hares were caught, collared and released are shaded in green lines and unsuccessful areas are shaded in pink lines. Area 1 (hares 8581 and 8582) was located along the centre of runway 16–34. Area 2 (hares 8583–8585) was located to the south‐east of the airfield. (b) E‐Obs fitted collar attached to hare 8584 prior to deployment and (c) Recorded GPS locations of hare 8484 (male) as an example. All GPS locations of all hares can be seen in the Supplementary Material (S1). *Photo credit: Gerry Keogh. Software: QGIS v 3.4, Basemap: OSM Standard*.

Dublin International Airport (53.4264° N, 6.2499° W) is the largest civil airport in Ireland, and with ~250,000 aircraft movements recorded in 2023 alone, it is also one of the busiest in Europe. The airfield is located approximately 7 km North of Dublin city and approximately 8 km from Irelands Eastern coastline, experiencing a temperate climate. There are three runways present at the airfield, only one of which (runway 10R‐28L; Figure 1a) was fully operational during the study period, due to ongoing construction works of runway 10L‐28R. Therefore, runway 10L‐28R was not operational at all during the study period with runway 16–34 being partially operational (~1.5 km). However, runway 16–34 was only sporadically active throughout the study period and constantly for taxiing aircraft from May 2022. Therefore, a tarmacked area was referred to as *‘active’* if it was operational for the entire study period, *‘semi‐operational’* if occasional movements/ taxiing occurred and *‘inactive’* if the area was closed to manoeuvring traffic. Surrounding the runways are fifty‐nine distinct islands of semi‐natural grassland, collectively ~3.4 km^2^ in size. A mean daily temperature of 10°C and daily precipitation of 46.5 mm was recorded throughout the study period (December 2021–August 2022; www.met.ie).

### 2.2 Data collection

Five adult Irish hares (*Lepus timidus hibernicus*) were captured using long nets in November 2021 from two locations at the airfield (Figure 1a). Approximately 100 m of nylon nets, each around 80 cm high, were set out for each catch attempt (*n* = 8 ‘walks’) with 10 beaters driving hares towards the nets on foot. Beaters were positioned approximately 10 m from each other, and made noise (clapping, vocal sounds) to encourage movement of the hares. Due to access restrictions associated with having personnel in close proximity to an active runway and near sensitive equipment (e.g. Instrument Landing System (ILS)), areas where netting attempts were permitted were limited (Figure 1a). Hares were sexed, (4 male, 1 female), weighed and fitted with a GPS collar (Model 1A Light, e‐obs GmbH, Gruenwald, Germany), which also featured a tri‐axial accelerometer. Handling time took between 10 and 15 min per hare, with hares released from where they had been captured (Figure 1b; Table 1). Collars weighed 65 g, which was ≤2% of captured hares' body weights (3.2–3.5 kg). GPS collars were programmed to record movement 24 h a day, but the GPS fix schedule depended on hare activity levels. As devices were accelerometer informed, a GPS fix was taken every 10 min when a minimum threshold of variance (>5000; manufacturer specific measurement) was met indicating an active animal, to capture fine scale movements. During periods of low activity (i.e. resting; below accelerometer variance threshold), a GPS fix was taken every 6 h to conserve battery. All hare capturing, handling and collaring were approved by the institute's (*Anonymous*) ethics committee (#2019–003) and national governing body (NPWS; licence numbers 01/2020 and C32/2020). The first 5 days of data were excluded from analysis to avoid the inclusion of possibly uncharacteristic movements influenced by catching and handling (Mayer et al., 2021).

**TABLE 1:** Summary of biometrics, data collection dates and number of fixes for each collared hare at Dublin Airport. See Figure 1a for location polygons.

Hare ID	Sex	Weight (kg)	Location	Data collection start	Data collection end	Reason	No. of days	No. of fixes	No. of tarmacked area interactions	% of fixes interacting with tarmacked area
8581	M	3.4	Area 1	05/12/2021	19/07/2022	Battery died	226	17,764	2239	12.6
8582	M	3.2	Area 1	05/12/2021	18/06/2022	Battery died	195	18,829	1798	9.5
8583	M	3.2	Area 2	05/12/2021	06/07/2022	Battery died	213	19,513	164	0.8
8584	M	3.2	Area 2	05/12/2021	10/07/2022	Battery died	217	19,926	341	1.7
8585	F	3.5	Area 2	05/12/2021	22/08/2022	Project end	260	15,650	29	0.2

### 2.3 Data processing

Data were downloaded every 2 weeks via remote download, using a portable base station and yagi antennae. These data were collected in Coordinated Universal Time (UTC) and converted to local time to account for daylight savings (27/03/2022 – study end) for analysis. We removed GPS fixes with a horizontal GPS fix accuracy >30 m to reduce the inclusion of false crossing events as taxiway and runway widths are ~35 and 60 m wide respectively at the airfield, removing 2% of GPS fixes. Tarmacked area interactions were defined as the linear intersections of the path connecting two consecutive GPS fixes and the tarmacked part of the airfield where aircraft are moving for the purpose of commencing, or completing an aircraft manoeuvre (i.e. take‐off or landing). These areas were categorised as ‘runways’ (defined as a rectangular area for the landing and take‐off run of aircraft), ‘taxiways’ (defined as the main routes running parallel to runways to allow aircraft to taxi to/from a runway) and ‘connecting areas’ (defined as minor routes connecting taxiway and runway networks). Intersections were determined in QGIS (V 3.4.4) using the ‘*Explode Lines’* and *‘Intersection’* processing tools. As a single movement track could intersect more than one tarmacked area, which represents an individual strike risk, each intersection was retained and was treated as an independent event. Each event was counted to determine the daily number of intersections per hare.

Hares generally exhibit a bimodal, crepuscular activity pattern (Caravaggi et al., 2018; Pettigrew et al., 2021), and this has also been recorded for this specific population (Ball et al., 2022). However, day length in Ireland varies substantially with the seasons, with the longest days in the summer recording ~17 h of daylight and the shortest days in winter ~7 h. Therefore, understanding how tarmacked area interactions vary with the rising/setting of the sun could have important management implications. To investigate this relationship, each tarmacked area interaction was offset to either the time of sunrise, or sunset (sourced: www.timeanddate.com), depending on when the interaction event occurred. All tarmacked area interactions, which occurred between the hours of 00:00 and 11:59 were offset relative to sunrise, and all events that occurred between 12:00 and 23:59 were offset relative to sunset (as per Caravaggi et al. (2018) & Ball et al. (2022)). All events which occurred between sunrise and sunset were converted to positive integers to indicate hours of daylight and all events which occurred between the hours of sunset and sunrise were converted to negative integers to indicate hours of darkness. As an example, on a day where sunrise was at 08:00 and sunset at 19:00, a detection at 11:30 would be offset relative to sunrise and given a value of +3 h 30 min. On the same day, an event that occurred at 21:00 would be assigned a value of −2 h. These offset values were used as the dependant variable in a Kruskal–Wallis, to test differences in activity patterns across seasons. Seasons were defined as winter (December–February), spring (March–May) and summer (June–August), with no available data for autumn (September–November) due to the end of the battery life of GPS tags (Table 1).

The average daily and cumulative monthly movements were calculated according to the total length of each track, to identify movement patterns in relation to month (*MoveApps*; Scharf, 2022). For monthly data, only those months where data were collected for the entire calendar month were included. To evaluate trends in daily movement across months, we used non‐parametric Kruskal–Wallis tests with a Dunn's post‐hoc test with a Benjamini–Hochberg *p*‐value correction (Benjamini & Hochberg, 1995), allowing for multiple comparisons, as parametric modelling assumptions were not met. Here, data for July were removed as they were only available for a single individual (8585).

To investigate the movement behaviour of the hares at the airfield, the Kernel Utilisation Distribution (KUD) of each individual was examined, with the reference value (h_ref_) selected as the smoothing parameter. This parameter considers the standard deviation of the x and y coordinates in addition to the number of relocations. The KUD is the probability density of relocating the animal at a specific location. Home ranges were extracted with 95% contour values, indicating the minimum area of which the probability of relocating the individual is 0.95 (95%). Data were processed using the ‘*adehabitatHR*’ package (Calenge, 2006) in R v. 4.0.4 (R Core Team, 2021). Habitat types were identified as ‘*Tarmacked areas’* (all surfaced parts of an airfield allowing for the movement of aircraft. This incorporates all runways, taxiways and the small connecting strips which connect the two, regardless of whether they were open to routine air traffic), ‘*Apron*’ (concrete/paved areas used for the parking, loading and fuelling of aircraft), ‘*Built land*’ (any artificial surfaces present for the purpose of facilitating airside services, such as the fire station, sub‐stations etc.), ‘*Grasslands*’ (airside areas consisting of open grass), ‘*Service roads*’ (airside road network which allows for vehicle access and movement) and ‘*Unknown’*, for instances where the habitat type could not be determined. The proportion of each habitat type within each home range was determined using the ‘*Overlap Analysis’* tool in QGIS. Step length was determined by calculating the distance between consecutive relocations for each individual.

## 3 RESULTS

### 3.1 Tarmacked area interactions

**FIGURE 2:**
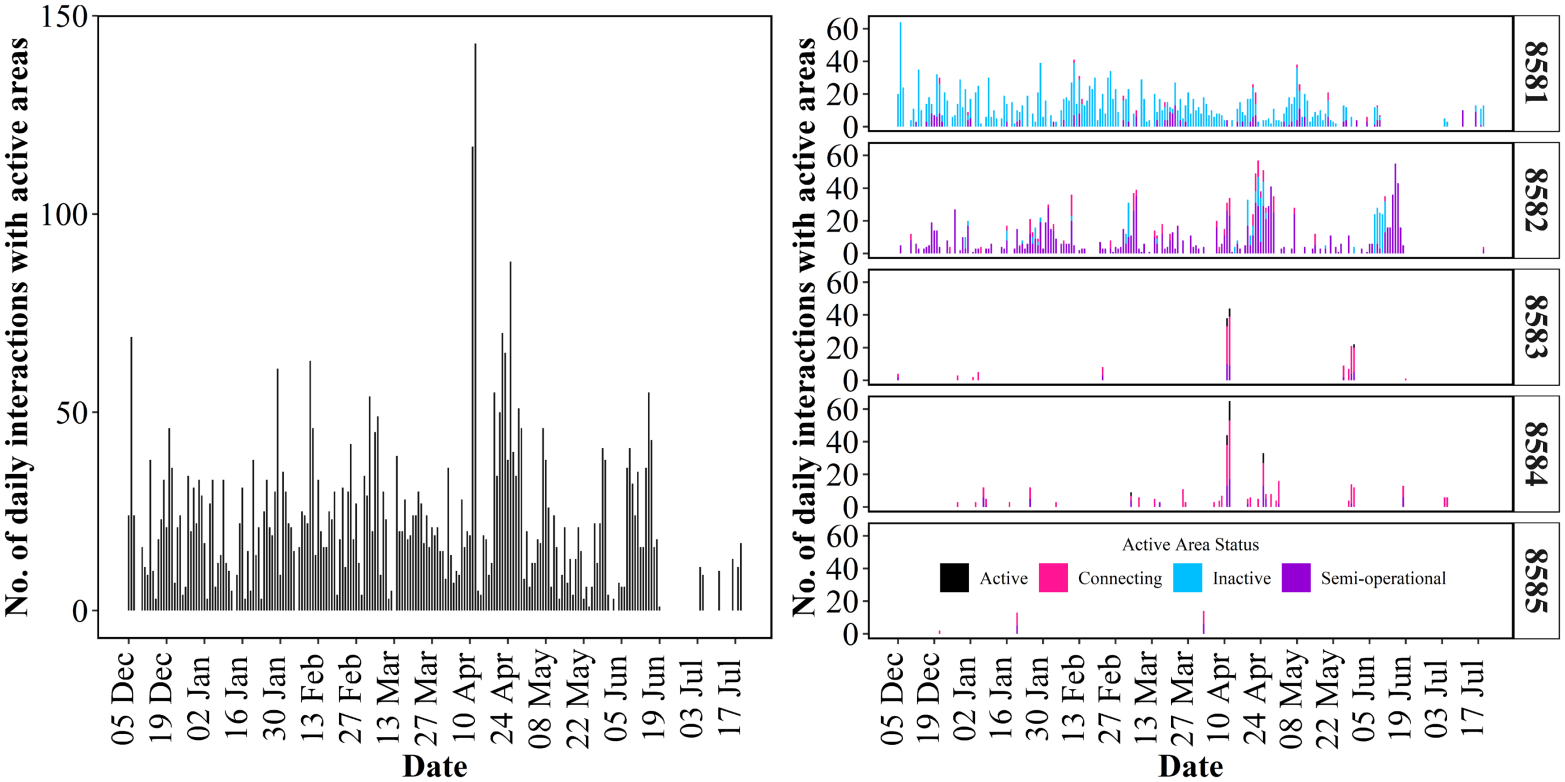
Number of tarmacked area interactions recorded daily for the duration of the study period for (*left*) all hares and (*right*) individual hares. Active = Runway and taxiway 10R‐28L open to air traffic; Semi‐Operational = Runway and taxiway 16–34 partially open to manoeuvres and to taxiing traffic from May 2022; Connecting = small areas, which connect runways to taxiways. These are grouped together as the activity status of each area changed with moving construction works. Therefore, activity of each area was not always known throughout the study period; Inactive = Runway and taxiway 10L‐28R not open to air traffic; Data collection commenced on December 5th 2021 and the last date an interaction was recorded was the 19th July 2022. Gaps indicate days where no interaction events were recorded. Dates are provided at 2‐week intervals. Hares 8581–8584 were males. Hare 8585 was female.

Our final dataset consisted of 91,682 GPS locations with an average horizontal error of 7.5 m and a total of 4571 tarmacked area interactions overall, spanning from December 5 to August 22 (Table 1). Average step‐length (based on GPS fixes from 10‐min intervals) across all hares was 54 m (±SD 83.3), with the highest average step‐length between consecutive relocations at 68.5 m (±SD 97.5) recorded for hare 8584 (male) and the lowest at 36.5 m (±SD 55.7) for hare 8585 (female). Across all individuals, an average of 5% (range = 0.2%–12.6%; Table 1) of movements (paths) included an interaction with a tarmacked area. There was a mean of 4.3 (range = 0–65) tarmacked area interactions, per hare, per day throughout the study period, with variation in the number of daily events recorded (Figure 2). Interactions were recorded for all months, with the highest mean number of interactions per hare, per month recorded for April (214.6; range = 14–531; Table 2).

**TABLE 2:** Total and mean number of interactions recorded for each month with tarmacked areas. Range indicates the lowest and highest number of interactions recorded for the month across individuals. Data for July are excluded.

Month	Total	Mean	Range
December	583	116.6	2–421
January	595	119	7–330
February	654	130.8	0–467
March	702	140.4	0–379
April	1073	214.6	14–531
May	502	100.4	0–295
June	391	78.2	0–339

Here, it is important to note that 88% of tarmacked area intersections involved the two male individuals from Area 1 (8581 and 8582), where there were limited aircraft movements on runways 16–34 (semi‐operational) and 10L‐28R (inactive) during the study period. Approximately a third of tarmacked area interactions intersected runways (37.3%; *n* = 1706), with 91.7% of runway interactions occurring with runway 16–34 (*n* = 1564; semi‐operational), 6.8% (*n* = 116) with runway 10L‐28R (inactive) and 1.5% (*n* = 26) with runway 10R‐28L (active). The largest proportion of interactions occurred with taxiways (54.7%; *n* = 2502), with 84.5% of taxiway interactions occurring with Taxiway 10L‐28R (*n* = 2120), followed by the taxiways and connecting links associated with runways 16–34 (14.8%; *n* = 370) and 10R‐28L (0.7%; *n* = 18). All remaining interactions (8%; *n* = 363) occurred with small sections of connecting areas.

**FIGURE 3:**
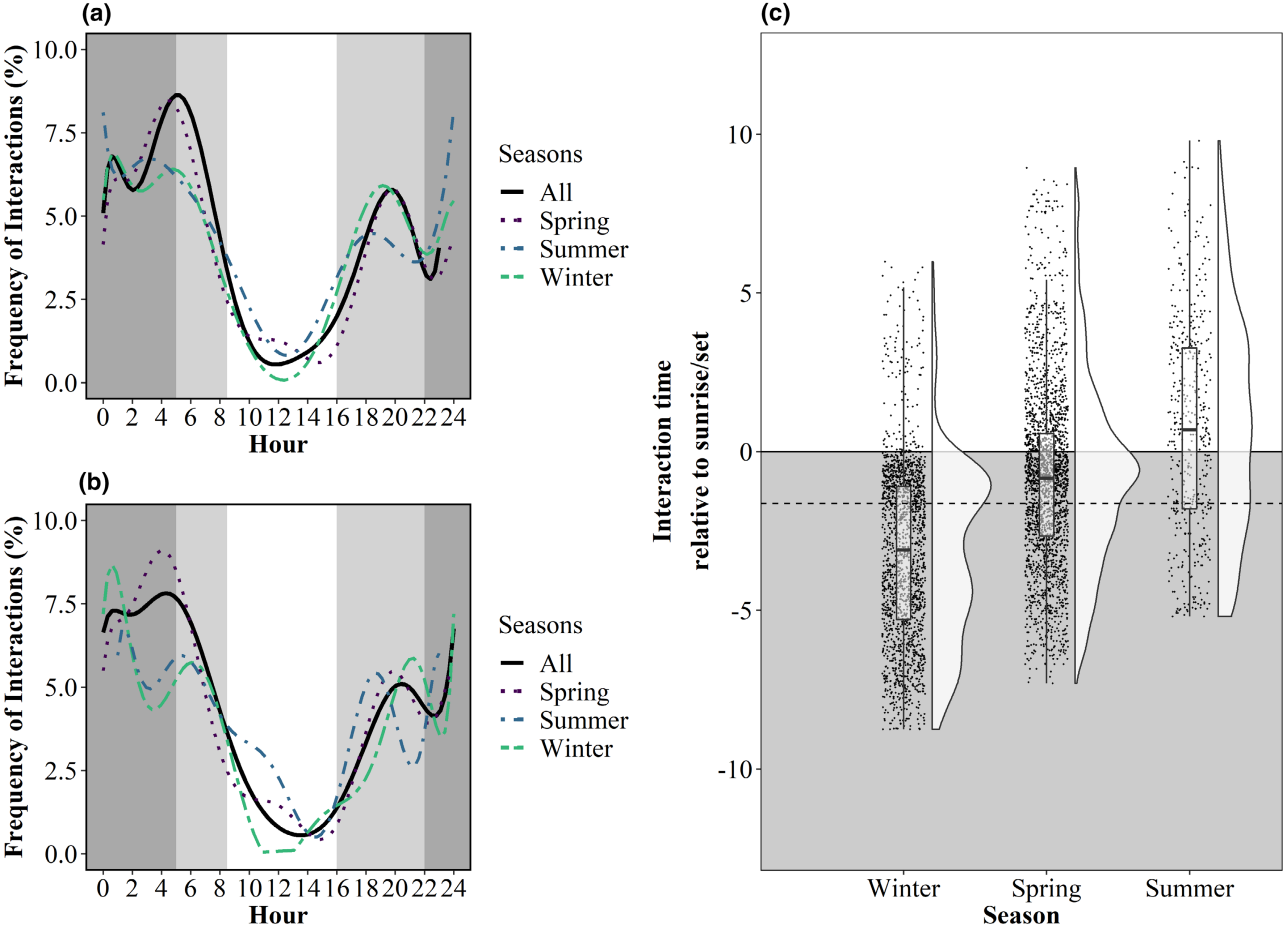
Temporal interactions of Irish hare with (a) all tarmacked areas and (b) runways (active, inactive and semi‐operational) only at Dublin Airport (December 2021–August 2022). Seasons were defined as Winter (Dec–Feb), Spring (Mar–May) and Summer (Jun‐Aug). Light grey shaded areas show the range of sunrise and sunset times throughout the study (i.e., 21st June vs. 21st December). Dark grey shaded areas show hours of darkness across the entire study. Interactions of Irish hare for all tarmacked area interactions relative to sunrise/sunset are shown in (c). Shaded areas imply hours after sunset and lighter areas imply hours after sunrise. Mean ± IQR are represented by boxplots (left) and the density and spread of the data are represented by raincloud plots (right; Allen et al., 2021). The mean annual offset of events relative to sunrise and sunset across all seasons is denoted by the dashed line.

Throughout the entire study period, interactions with tarmacked areas peaked between 05:00 and 07:59 (24% of interactions; Figure 3a), with some variation between seasons, particularly for the summer months when activity peaked between 00:00 and 03:59 (28%). Over half of runway interactions took place between 00:00 and 07:59 (57.5%), with a peak between 03:00 and 05:59 (23%; Figure 3b). Significant differences (*p* < .001) in tarmacked area interactions relative to sunrise/sunset were recorded across all seasons at Dublin Airport (*X*_2_, 801 = 2, *p* < .0001), with interactions in the summer occurring predominately in hours of daylight, particularly within the hour following sunrise and preceding sunset. In comparison, interactions in the spring and winter mainly occurred during hours of darkness, with those in the spring occurring within 2 h prior to sunrise and within 2 h of sunset. Winter interactions mainly occurred during hours of darkness, with relatively few interactions following sunrise or preceding sunset. Offset values relative to sunrise and sunset (Figure 3) indicate that a high proportion of interactions occurred within the first 2 h following sunset and preceding sunrise (33% of detections) across all seasons (Figure 3c).

### 3.2 Daily movements

**FIGURE 4:**
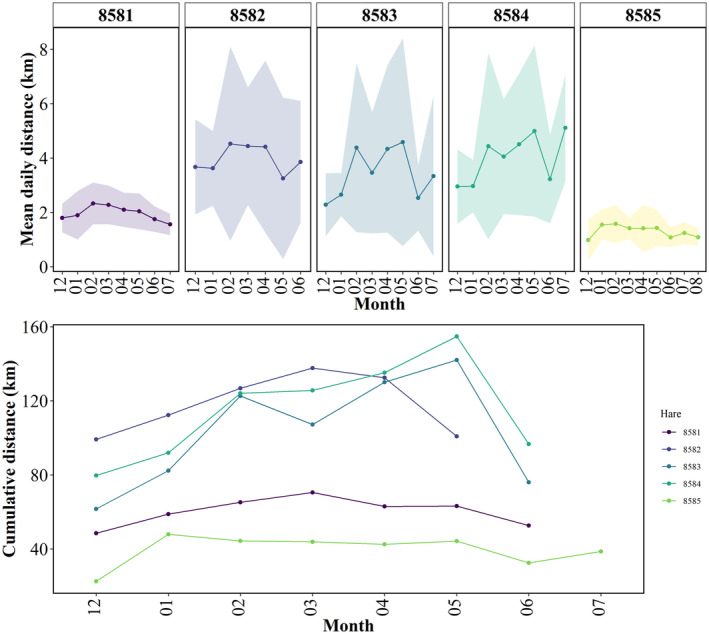
(Top) Mean(±SD) daily distance covered by each hare according to month (December–August). As daily data were used, all data (including for incomplete months) were used. (Bottom) Cumulative distance (sum of all daily distances) covered by each hare according to month. As several collars stopped recording part‐way through a month, only months where collars recorded for the entire calendar month were included with exception for December (12) where all data collection commenced on 05.12.21. Hares 8581–8584 were males. Hare 8585 was female.

Daily movements varied between individuals across the study period (Figure 4). The mean daily distance travelled by all hares throughout the study period was 2.87 km/ day (*±*SD 1.57 km), with greatest mean daily distance recorded for the month of February (3.45 km; *±*SD 2.90 km). Collective cumulative distance (sum of all daily distances) was greatest for April (503 km; x¯ = 3.35 km; *±*SD 2.67 km) and May (505 km; x¯ = 3.26 km; *±*SD 2.93 km). Daily movements were generally lowest during December–January, increasing over the following months, and declining in May–June. There were significant differences in daily distances travelled according to month (H(6) = 31.5, *p* = <0.001), with June being significantly different to all other months with the exception of December, as shorter daily distances were recorded for the months of December and June. Significant differences were also recorded for December for all months between January and April inclusive (Supplementary Material S2).

### 3.3 Airfield utilisation

**FIGURE 5:**
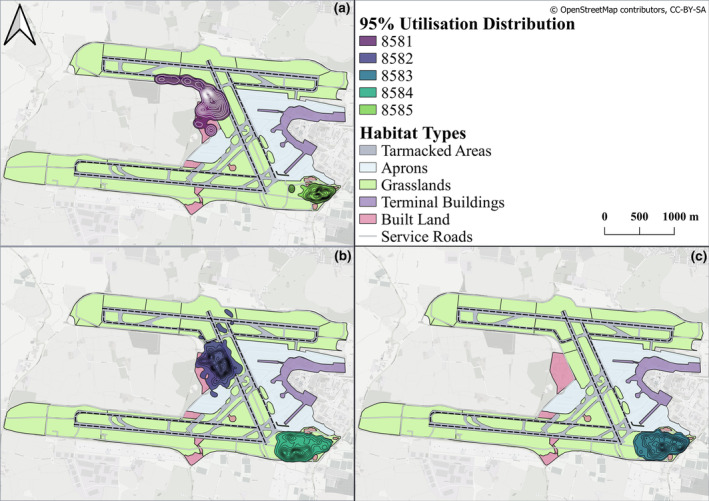
Home ranges of the five collared hares at Dublin Airport based on 95% Kernel Utilisation Distribution. (a) Hares 8581 (male) and 8585 (female). (b) Hares 8582 (male) and 8584 (male) and (c) Hare 8583 (male).

Home range sizes varied between individuals, ranging from 0.1 km^2^ (8585) to 0.4 km^2^ (8581), with an average home range size of 0.28 km^2^ (±SD 0.1 km^2^; Table 3; Figure 5). As previously defined, ‘*Tarmacked Areas’* are considered to be all runways, taxiways and small connecting corridors linking the two, regardless of whether they were open to routine air traffic or not. The home ranges of only two individuals (8581; 8582) incorporated these tarmacked areas, with the home range of 8582 incorporating runway 16–34 and the home range of 8581 incorporating both runway 16–34 and Taxiway 10L‐28 (Figure 5), both of which were not open to routine air traffic during the time of data collection, classified as ‘semi‐operational’ and ‘inactive’, respectively. The home ranges of the three remaining hares did not incorporate any tarmacked areas, but hares 8583 and 8584 both entered the first 110 m (approximate) of the main runway (10R‐28L), which was open to routine air traffic during the study period (i.e., active).

**TABLE 3:** Home range size (ha) estimated for each collared hare estimated with 95% Kernel Utilisation Distribution and the proportion (%) of each habitat type within each home range.

Hare ID	Sex	Home range size estimate (ha)	Tarmacked area*	Apron	Built land	Grassland	Service roads	Unknown^†^
8581	M	36.0	16%^^^	1%	28%	47%	3%	5%
8582	M	33.6	10.5%^^^	6%	28%	53%	1.5%	1%
8583	M	27.6	0%	1%	5%	87%	7%	0%
8584	M	28.7	0%	0.5%	4%	89%	6.5%	0%
8585	F	12.2	0%	0%	9%	86%	5%	0%

* Denotes all (active, semi‐operational and inactive) runway/ taxiway/connecting habitat types.

^^^ Denotes that tarmacked areas used were either inactive or semi‐operational at time of study (runway 10L‐28R & taxiway 10L‐28R).

^†^ Denotes area of estimated home range (95% KUD) that falls outside of the airfield boundary into neighbouring agricultural grassland. Usage is likely to be a combination of this grassland and airside habitat usage directly adjacent to the airfield perimeter fence.

Despite the presence of the boundary fence around the airfield, hares 8581 and 8582 were recorded outside of the airfield perimeter in proximity to the airfield (e.g. staff carparks, security posts). There was a single incident of a hare (8582) crossing the regional road running parallel to the airfield and entering the surrounding farmland before returning to the airfield via a security check barrier approximately 6 h later. This gatepost allows for the entry and exit of heavy goods vehicles and personnel.

## 4 DISCUSSION

Identifying patterns as to when high‐risk species are entering tarmacked areas is essential to the development of strike mitigation measures on both a seasonal and temporal scale. Tarmacked area interactions occurred during the current study according to a bimodal, crepuscular pattern, similar to activity patterns previously recorded for Irish hares (Caravaggi et al., 2018) and indeed, for this specific population (camera trap data; Ball et al., 2022). We found cumulative movement and the number of tarmacked airfield interactions to be highest during the Spring sampling period. The cumulative movement travelled between points is likely to be a conservative estimate as, distance estimates were based on linear movements and animals often adopt more complicated movement trajectories (e.g., zig‐zag; Spitz & Janeau, 1990). The two months where the highest monthly cumulative movements were recorded (April and May) coincided with the period at the airfield with the highest proportion of historic strike events (21% of all strike events, 2012–2022; *pers comms*; Ball, Butler, et al., 2021). It should be noted, however, that there is no discernible pattern to strikes at Dublin Airport as strike events occur year‐round (Ball, Butler, et al., 2021).

The high cumulative number of movements recorded for hares during the spring (March–May) likely coincides with increased activity levels associated with Irish hare mating behaviour. The Irish hare has a prolonged breeding period, potentially breeding year‐round in favourable conditions (Byrne et al., 2022). Indeed, leverets were observed in all seasons at the airfield (*SB*, *pers obs*) and collared hares were observed mating in February (8582) and May (8584 and 8585). However, high energy breeding displays – characterised by groups of males chasing a female and the famed ‘boxing’ displays – are typically observed at the airfield between March and May. During these displays, hares can be observed travelling at high speed, crossing through grasslands, service roads and tarmacked areas (*SB*, *pers. obs*). Such breeding behaviour may explain the high number of recorded tarmacked area interactions on April 11th–12th by two hares that typically did not frequent such areas (8583 and 8584) and may have been pursuing the same female. This could indicate that breeding activities, particularly in the spring, increase tarmacked area interactions and therefore strike risk in this population. Indeed, a higher incidence of hare‐road interactions and mortalities have been recorded for hare species (*L. europaeus and L. timidus*) in the spring, likely due to mating behaviours (Raymond et al., 2021, Mayer et al., 2022).

Understanding the frequency with which animals are engaging with tarmacked areas can allow for the quantification of strike risk. Throughout the study period, the frequency with which hares interacted with tarmacked areas varied between individuals, with those located in close proximity to runway habitats (8581 and 8582) utilising tarmacked areas in up to 13% of their relocations. We recorded an average of 4.3 tarmacked area interactions per hare, per day. As the population is estimated at ~118 individuals (*SB*, *Unpublished data*), this could translate to an approximate estimate of 507.4 tarmacked area interactions daily across the population. However, with the majority of interactions from these data having been recorded with semi‐operational or inactive areas (runways 16–34 and 10L‐28R, respectively), it is uncertain how frequently hares would interact with tarmacked areas with frequent aircraft movements. At Dublin Airport, aircraft movements increase from c.06:00 and remain constant for the duration of the day, before dropping off approaching midnight (00:00; Ball et al., 2022).

Observations of hares interacting with runway 10L‐28R, prior to becoming active, have highlighted that hares spend an average of 1 min and 7 s on runways and taxiways (*n* = 31, S.B., *Unpublished data*). On two instances, hares were observed resting on the inactive runway for 16 min 11 s and 9 min and 13 s, which is unlikely to occur when the runway is experiencing aircraft manoeuvres. With these two observations removed, the duration of time a hare spent in a tarmacked area is an average of 19 s (±SD 17 s). However, across the entire population, this relatively short duration of time becomes substantial (9633 s, or 2.6 h assuming non‐concurrent interactions).

In the current study, locations were recorded every 10 min to ensure that data could be collected over multiple months. While it is therefore likely that tarmacked area interactions were missed (e.g. where hares may have intersected these areas and returned to the original side of an area in <10 min), average step length of relocations (54 m) was less than the width of the runways present at Dublin Airport (>60 m). Therefore, we are confident that these data are an accurate representation of the hares' interactions with tarmacked areas.

We were unable to explicitly demonstrate if hares actively avoided active, tarmacked areas, as a delay in the opening of new runway (10L‐28R) by several months meant that batteries had expired in the two collars of individuals (8581, 8582) in proximity to the new runway. Hares 8581 and 8582 incorporated 534 m and 395 m of runway 16–34 into their home ranges, with hare 8581 also incorporating 1004 m of taxiway 10L‐28R. However, it is unknown whether these home ranges would have been maintained or shifted with the opening of runway 10L‐28R in late August 2022 due to increased levels of disturbance. Likewise, for the three collared hares in proximity to the main active runway (10R‐28L), the main runway was outside of their regular home range with a limited number of interactions. While this may indeed be indicative of active area avoidance, given the average home range size of the collared hares (0.28 km^2^), there are few locations around the airfield where it would be possible to completely exclude all active areas from an individual's home range, with ‘*Area 2*’ (Figure 1a.) being one such area (Figure 1c). The home range sizes we recorded for the Irish hare population at Dublin Airport were similar to those reported elsewhere in Ireland (Caravaggi et al., 2016; Reid, 2023; Wolfe & Hayden, 1996). Hares do not inherently avoid tarmacked surfaces, however. For example, it has been demonstrated that European hares (*L. europaeus*) incorporate main roads into their home ranges (Mayer et al., 2022) and road interactions increase in homogenous landscapes to meet individual environmental requirements (e.g. food, shelter). Hence, it is feasible that hares avoid active areas when inhabiting an area large enough to meet environmental requirements and that busy, active areas are utilised and are incorporated into individuals home ranges when needed.

It is important to consider that the application of GPS and other, similar devices may be not deemed appropriate or permissible for use in many airfields due to concerns relating to potential strike events with animals carrying battery operated biologgers. Strikes involving animals the size and body mass of a hare can cause substantial damage (Ball, Butler, et al., 2021) and the addition of non‐organic equipment could have additional consequences for aircraft involved. Indeed, this was the first known study to deploy biologgers onto a free roaming, airside wildlife population. However, there has been a rapid increase in the rate of GPS tracking studies conducted in recent years, with much of the data available on accessible platforms (e.g. Movebank). These studies, conducted away from airfields, could allow for insights into habitat selection, spatial use and behaviours of hazardous species, which could help to inform airside wildlife hazard management. For example, Arrondo et al. (2021) utilised available data detailing flight patterns and height of bird species involved in strike events in Spain, to predict hazards to aircraft. Similarly, data for hazardous mammal species could be utilised, to identify likely periods of increased risk (e.g. Ball et al., 2022). Likewise, it is important to note that alternative remote sensing methods (e.g. camera traps) may be a more appropriate option for airfield managers, depending on the type of data required. For example, in this study, the times in which GPS collars recorded interaction events with tarmacked areas (runways/taxiways) were similar to circadian activity data captured from motion activated camera traps (Ball et al., 2022).

Air transportation is a fundamental component of modern society, with passenger numbers having increased by almost 350% from 1990 to 2019, prior to the COVID‐19 pandemic (www.worldbank.org). Given this upward trajectory, it is likely that mammal strikes will become increasingly common and hazardous to aviation, in addition to having negative impacts for wildlife and conservation efforts (Ball, Caravaggi, & Butler, 2021). Here, we demonstrate the application of GPS tracking devices and their suitability for identifying potentially hazardous movement patterns within an airfield environment, indicating when animals are likely to come into contact with aircraft. At Dublin Airport, there has been an average of 0.55 ± 0.12 strikes per 10,000 aircraft movements over 30 years, peaking at 1.89 strikes per 10,000 movements in 2018 (Ball, Butler, et al., 2021). Our data show considerable individual variation in movement behaviour and add to existing evidence on periods of increased strike risk at Dublin airport. The novel insights gained from this study will lead to improved management plans aimed at mitigating strike risk. This study also serves as an important first‐step towards studying airside mammal populations, one that we hope will inspire further collaborations between researchers and authorities, worldwide, and the development of species‐ and airport‐specific management plans.

## AUTHOR CONTRIBUTIONS

**Samantha Ball:** Conceptualization (lead); data curation (lead); formal analysis (lead); investigation (lead); methodology (equal); visualization (lead); writing – original draft (lead); writing – review and editing (lead). **Anthony Caravaggi:** Formal analysis (supporting); funding acquisition (supporting); methodology (supporting); supervision (supporting); writing – original draft (supporting); writing – review and editing (supporting). **Gerry Keogh:** Funding acquisition (lead); project administration (supporting); resources (supporting); supervision (supporting). **Fidelma Butler:** Funding acquisition (supporting); project administration (lead); supervision (lead); writing – original draft (supporting); writing – review and editing (supporting).

## FUNDING INFORMATION

This research was funded collaboratively (EBPPG/2018/43) by the Irish Research Council (IRC), University College Cork (UCC) and Dublin Airport (daa).

## CONFLICT OF INTEREST STATEMENT

The lead author (SB) was employed for the duration of the project by Dublin Airport for the sole purpose of conducting research.

## Supporting information


Data S1.

## Data Availability

Data are available on Movebank (Movebank ID: 1861050141) and Figshare. Due to the sensitive nature of the data, data access is restricted but can be requested from the authors. The link to the study can be found at: https://www.movebank.org/cms/webapp?gwt_fragment=page=studies,path=study1861050141.

## References

[ece311490-bib-0001] Allen, M. , Poggiali, D. , Whitaker, K. , Marshall, T. R. , van Langen, J. , & Kievit, R. A. (2021). Raincloud plots: A multi‐platform tool for robust data visualization. Wellcome Open Research, 4, 63. 31069261 10.12688/wellcomeopenres.15191.1 PMC6480976

[ece311490-bib-0002] Arrondo, E. , García‐Alfonso, M. , Blas, J. , Cortes‐Avizanda, A. , De la Riva, M. , Devault, T. L. , Fiedler, W. , Flack, A. , Jimenez, J. , Lambertucci, S. A. , Margalida, A. , Oliva‐Vidal, P. , Phipps, W. L. , Sanchez‐Zapata, J. A. , Wikelski, M. , & Donazar, J. A. (2021). Use of avian GPS tracking to mitigate human fatalities from bird strikes caused by large soaring birds. Journal of Applied Ecology, 58, 1411–1420.

[ece311490-bib-0003] Askren, R. J. , Dorak, B. E. , Hagy, H. M. , Eichholz, M. W. , Washburn, B. E. , & Ward, M. P. (2019). Tracking Canada geese near airports: Using spatial data to better inform management. Human‐Wildlife Interactions, 13, 344–355.

[ece311490-bib-0004] Avisure . (2022). Fatalities and Destroyed Aircraft due to Wildlife Strikes. https://avisure.com/wp/serious‐accident‐database/

[ece311490-bib-0005] Ball, S. , Butler, F. , Caravaggi, A. , Coughlan, N. E. , Keogh, G. , Callaghan, M. J. A. O. , Whelan, R. , & Kelly, T. C. (2021). Hares in the long grass : Increased aircraft related mortality of the Irish hare (Lepus timidus hibernicus) over a 30‐ year period at Ireland's largest civil airport. European Journal of Wildlife Research, 67, 67–80.

[ece311490-bib-0006] Ball, S. , Caravaggi, A. , & Butler, F. (2021). Runway roadkill : A global review of mammal strikes with aircraft. Mammal Review, 51, 420–435.

[ece311490-bib-0007] Ball, S. , Caravaggi, A. , & Butler, F. (2022). Hareport hazard : Identifying hare activity patterns and increased mammal – Aircraft strike risk at an international airport. Remote Sensing in Ecology and Conservation, 2, 1–13.

[ece311490-bib-0008] Benjamini, Y. , & Hochberg, Y. (1995). Controlling the false discovery rate : A practical and powerful approach to multiple testing. Journal of the Royal Statistical Society, 57, 289–300.

[ece311490-bib-0009] Byrne, A. W. , Marnell, F. , Barrett, D. , Reid, N. , Hanna, R. E. B. , & Mcelroy, M. C. (2022). Rabbit Haemorrhagic disease virus 2 (RHDV2; GI.2) in Ireland focusing on wild Irish hares (*Lepus timidus hibernicus*): An overview of the first outbreaks and contextual review. Pathogens, 2, 1–17. 10.3390/pathogens11030288 PMC8953227 35335613

[ece311490-bib-0010] Calenge, C. (2006). The package “adehabitat” for the R software: A tool for the analysis of space and habitat use by animals. Ecological Modelling, 197, 516–519.

[ece311490-bib-0011] Caravaggi, A. , Gatta, M. , Vallely, M. , Hogg, K. , Freeman, M. , Fadaei, E. , et al. (2018). Seasonal and predator‐prey effects on circadian activity of free‐ranging mammals revealed by camera traps. Peer J, 6, 1–27. 10.7717/peerj.5827 PMC6252065 30498626

[ece311490-bib-0012] Caravaggi, A. , Zaccaroni, M. , Riga, F. , Schai‐Braun, S. C. , Dick, J. T. A. , Montgomery, W. I. , & Reid, N. (2016). An invasive‐native mammalian species replacement process captured by camera trap survey random encounter models. Remote Sensing in Ecology and Conservation, 2, 45–58.

[ece311490-bib-0013] Carswell, B. M. , Rea, R. V. , Searing, G. F. , & Hesse, G. (2021). Towards building a species‐specific risk model for mammal‐aircraft strikes. Journal of Airport Management, 15, 288–303.

[ece311490-bib-0014] Chan, Y. C. , Tibbitts, T. L. , Dorofeev, D. , Hassell, C. J. , & Piersma, T. (2022). Hidden in plain sight: Migration routes of the elusive Anadyr bar‐tailed godwit revealed by satellite tracking. Journal of Avian Biology, 2022, e02988.

[ece311490-bib-0015] Corriveau, A. , Klaassen, M. , Garnett, S. T. , Kaestli, M. , Christian, K. , Crewe, T. L. , Loewensteiner, D. A. , & Campbell, H. A. (2022). Seasonal space use and habitat selection in magpie geese: Implications for reducing human‐wildlife conflicts. Journal of Wildlife Management, 86, 1–21.

[ece311490-bib-0016] Dias, T. C. , Stabach, J. A. , Huang, Q. , Labruna, M. B. , Leimgruber, P. , Ferraz, K. M. P. M. B. , Lopes, B. , Luz, H. R. , Costa, F. B. , Benatti, H. R. , Correa, L. R. , Nievas, A. M. , Monticelli, P. F. , Piovezan, U. , Szabó, M. P. J. , Aguiar, D. M. , Brites‐Neto, J. , Port‐Carvalho, M. , & Rocha, V. J. (2020). Habitat selection in natural and human‐modified landscapes by capybaras (*Hydrochoerus hydrochaeris*), an important host for Amblyomma sculptum ticks. PLoS One, 15, 1–17. 10.1371/journal.pone.0229277 PMC7444575 32817698

[ece311490-bib-0017] Dolbeer, R. , Begier, M. J. , Miller, P. R. , Weller, J. , & Anderson, A. (2023). *Strikes to Civil Aircraft in the United States, 1990–2022*. Federal Aviation Administration. U.S. Department of Agriculture, Animal and Plant Health Inspection Service, Wildlife Services.

[ece311490-bib-0018] Lehrke, R. M. , McGregor, L. , Dyer, J. , Stanley, M. C. , & Dennis, T. E. (2017). An inexpensive satellite‐download GPS receiver for wildlife: Field trial on black swans. Wildlife Research, 44, 558–564.

[ece311490-bib-0019] Mayer, M. , Fischer, C. , Blaum, N. , Sunde, P. , & Ullmann, W. (2022). Influence of roads on space use by European hares in different landscapes. Landscape Ecology, 38, 131–146.

[ece311490-bib-0020] Mayer, M. , Haugaard, L. , & Sunde, P. (2021). Scared as a hare: Effects of capture and experimental disturbance on survival and movement behavior of European hares. Wildlife Biology, 2021, wlb‐00840.

[ece311490-bib-0021] Parra‐Torres, Y. , Ramírez, F. , Afán, I. , Aguzzi, J. , Bouten, W. , Forero, M. G. , & Navarro, J. (2020). Behavioral rhythms of an opportunistic predator living in anthropogenic landscapes. Movement Ecology, 8, 1–8. 32341783 10.1186/s40462-020-00205-x PMC7183138

[ece311490-bib-0022] Pettigrew, G. W. , Di Vita, V. , Pettigrew, M. , & Gilchrist, J. S. (2021). The diel activity pattern of mountain hare (*Lepus timidus*) on managed heather moorland in Scotland. Ecology and Evolution, 11, 7106–7113. 34188797 10.1002/ece3.7512 PMC8216900

[ece311490-bib-0023] R Core Team . (2021). R: A Language and Environment for Statistical Computing.

[ece311490-bib-0024] Raymond, S. , Schwartz, A. L. W. , Thomas, R. J. , Chadwick, E. , & Perkins, S. E. (2021). Temporal patterns of wildlife roadkill in the UK. PLoS One, 16, 1–17. 10.1371/journal.pone.0258083 PMC8494347 34613989

[ece311490-bib-0025] Reid, N. (2023). Survival, movements, home range size and dispersal of hares after coursing and/or translocation. PLoS One, 18, e0286771. 37267331 10.1371/journal.pone.0286771 PMC10237436

[ece311490-bib-0026] Scharf AK (2022) Distance moved. MoveApps version v1. https://github.com/movestore/DistanceMoved.git.

[ece311490-bib-0028] Serieys, L. E. K. , Rogan, M. S. , Matsushima, S. S. , & Wilmers, C. C. (2021). Road‐crossings, vegetative cover, land use and poisons interact to influence corridor effectiveness. Biological Conservation, 253, 108930.

[ece311490-bib-0029] Spitz, F. , & Janeau, G. (1990). Spatial strategies: An attempt to classify daily movements of wild boar. Acta Theriologica, 35, 129–149.

[ece311490-bib-0030] Suraci, J. P. , Frank, L. G. , Oriol‐Cotterill, A. , Ekwanga, S. , Williams, T. M. , & Wilmers, C. C. (2019). Behavior‐specific habitat selection by African lions may promote their persistence in a human‐dominated landscape. Ecology, 100, e02644. 30714129 10.1002/ecy.2644

[ece311490-bib-0031] Voigt, C. C. , Scholl, J. M. , Bauer, J. , Teige, T. , Yovel, Y. , Kramer‐Schadt, S. , & Gras, P. (2020). Movement responses of common noctule bats to the illuminated urban landscape. Landscape Ecology, 35, 189–201.

[ece311490-bib-0032] Wolfe, A. , & Hayden, T. J. (1996). Home range sizes of Irish mountain hares on coastal grassland. Biology and Environment, 96, 141–146.

