# Supplementary material for: Dublin Hareport: The movement ecology and airfield interactions of resident, airside hares, at an international airport
Source: Ecol Evol. 2024 May 30;14(6):e11490. doi: 10.1002/ece3.11490 (PMC11139675; doi:10.1002/ece3.11490)
Supplement: Supplementary file 1 — Data S1. [file ECE3-14-e11490-s001.docx]

**S1:** Recorded GPS locations for five collared hares at Dublin Airport spanning from December 5^th^ to August 22^nd^ **A)** Hares 8581 (male) and 8585 (female). **B)** Hares 8582 (male) and 8584 (male) and **C)** Hare 8583 (male).


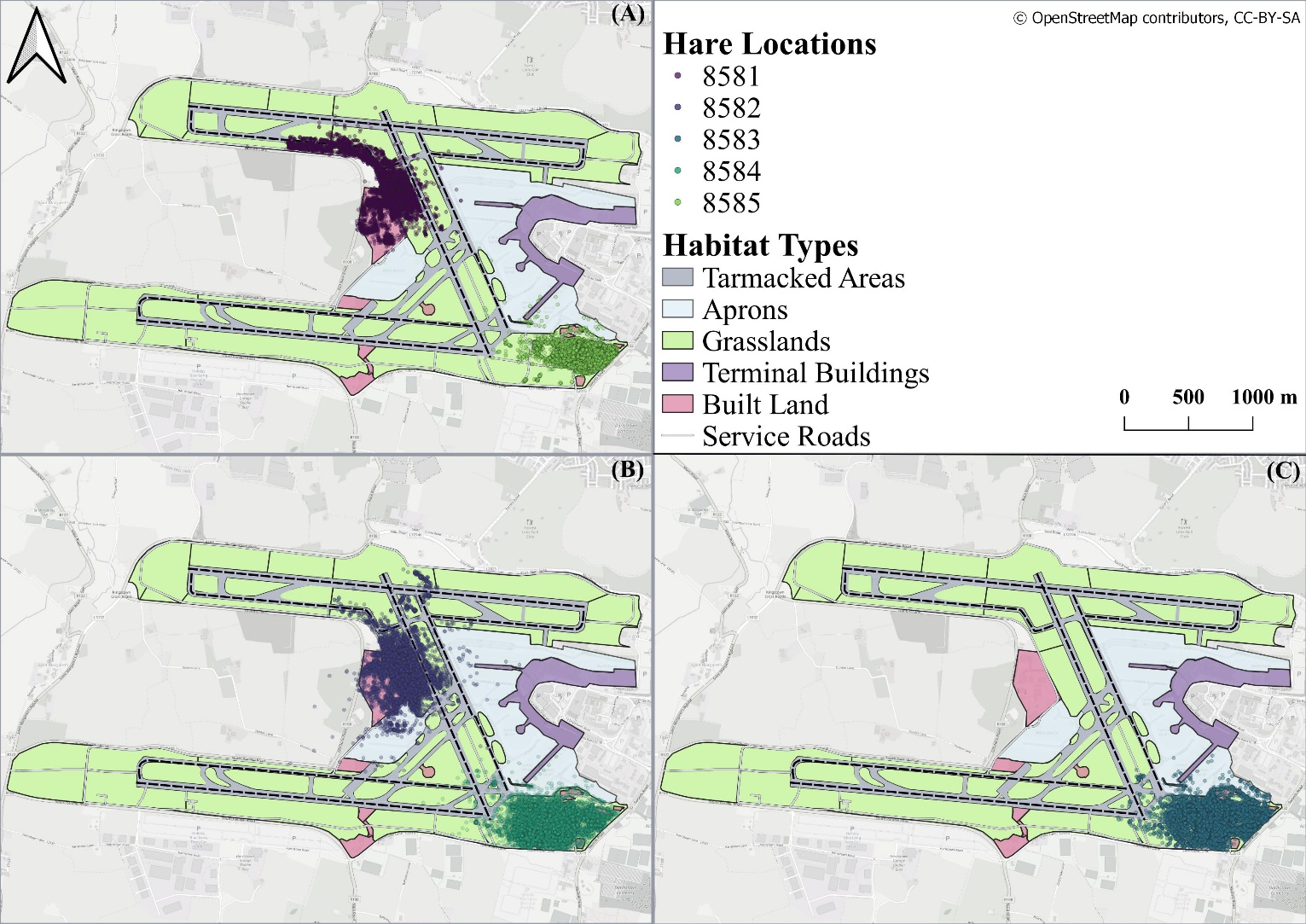


**S2**

Significance values with a Bonferroni Correction for differences in daily distances travelled (December- June). Significant values are indicated with an asterisk (*).

|  | December | January | February | March | April | May |
| --- | --- | --- | --- | --- | --- | --- |
| January | 0.3 | - | - | - | - | - |
| February | <0.05* | 0.3 | - | - | - | - |
| March | 0.008* | 0.1 | 0.8 | - | - | - |
| April | <0.05* | 0.3 | 0.9 | 0.8 | - | - |
| May | <0.05* | 0.8 | 0.2 | 0.2 | 0.3 | - |
| June | 0.3 | <0.01* | <0.001* | <0.0001* | <0.001* | 0.06 |
